# Polyphenols: Natural Preservatives with Promising Applications in Food, Cosmetics and Pharma Industries; Problems and Toxicity Associated with Synthetic Preservatives; Impact of Misleading Advertisements; Recent Trends in Preservation and Legislation

**DOI:** 10.3390/ma16134793

**Published:** 2023-07-03

**Authors:** Priyanka Rathee, Renu Sehrawat, Pooja Rathee, Anurag Khatkar, Esra Küpeli Akkol, Sarita Khatkar, Neelam Redhu, Gizem Türkcanoğlu, Eduardo Sobarzo-Sánchez

**Affiliations:** 1 Faculty of Pharmaceutical Sciences, Baba Mastnath University, Rohtak 124021, India; p.rathee85@gmail.com; 2 School of Medical and Allied Sciences, K.R. Mangalam University, Gurugram 122103, India; renusehrawat@gmail.com; 3 Department of Pharmaceutical Sciences, Maharshi Dayanand University, Rohtak 124001, India; poojarathee2108@gmail.com; 4 Department of Pharmacognosy, Faculty of Pharmacy, Gazi University, Ankara 06330, Turkey; gizemturkcanoglu@gazi.edu.tr; 5 Vaish Institute of Pharmaceutical Education and Research, Rohtak 124001, India; drsaritamdu@gmail.com; 6 Department of Microbiology, Maharshi Dayanand University, Rohtak 124001, India; neelamredhumajra@gmail.com; 7 Instituto de Investigación y Postgrado, Facultad de Ciencias de la Salud, Universidad Central de Chile, Santiago 8330507, Chile; 8 Department of Organic Chemistry, Faculty of Pharmacy, University of Santiago de Compostela, 15782 Santiago de Compostela, Spain

**Keywords:** natural phenolic compounds, preservatives, antioxidant, microbial activities, radical scavenging

## Abstract

The global market of food, cosmetics, and pharmaceutical products requires continuous tracking of harmful ingredients and microbial contamination for the sake of the safety of both products and consumers as these products greatly dominate the consumer’s health, directly or indirectly. The existence, survival, and growth of microorganisms in the product may lead to physicochemical degradation or spoilage and may infect the consumer at another end. It has become a challenge for industries to produce a product that is safe, self-stable, and has high nutritional value, as many factors such as physical, chemical, enzymatic, or microbial activities are responsible for causing spoilage to the product within the due course of time. Thus, preservatives are added to retain the virtue of the product to ensure its safety for the consumer. Nowadays, the use of synthetic/artificial preservatives has become common and has not been widely accepted by consumers as they are aware of the fact that exposure to preservatives can lead to adverse effects on health, which is a major area of concern for researchers. Naturally occurring phenolic compounds appear to be extensively used as bio-preservatives to prolong the shelf life of the finished product. Based on the convincing shreds of evidence reported in the literature, it is suggested that phenolic compounds and their derivatives have massive potential to be investigated for the development of new moieties and are proven to be promising drug molecules. The objective of this article is to provide an overview of the significant role of phenolic compounds and their derivatives in the preservation of perishable products from microbial attack due to their exclusive antioxidant and free radical scavenging properties and the problems associated with the use of synthetic preservatives in pharmaceutical products. This article also analyzes the recent trends in preservation along with technical norms that regulate the food, cosmetic, and pharmaceutical products in the developing countries.

## 1. Introduction

Shelf life refers to the duration between manufacturing and expiry date, during which the product is expected to retain its original characteristics and remain acceptable for consumers as far as its quality is concerned. During this duration, the product is susceptible to chemical, biological, and physical deterioration, which ultimately degrade the qualitative characteristics of the product [1]. Therefore, preservatives have been extensively used in various pharmaceuticals, cosmetics, and food products to prevent them from deterioration [2,3]. Many factors are responsible for governing the mean life of the product such as the growth of microorganisms, heating, inappropriate temperature, long storage, change in moisture content, reaction with light and oxygen, fermentation, acidification, enzymatic changes, etc., which result in the loss of important properties of the finished product. Pharmaceutical preparation consists of a diverse range of structures and moieties that are susceptible to deterioration. Deterioration is the result of chemical reactions that occur between the various ingredients present in the formulation and the external environment. The deterioration of a product generally occurs during longer storage, affecting its stability, ultimately resulting in the product’s decline in its intended natural quality due to microbial contamination and rendering the product harmful to the consumer. In general, there are three processes by which the product degrades [4]. Chemical breakdown includes chemical incompatibilities, such as hydrolysis, oxidation, photolysis, polymerization, hydration, dehydration, and decarboxylation. [5,6]. A change in temperature, particle size, evaporation, vaporization, efflorescence, hygroscope, deliquescence, etc. are all examples of physical degradation. Everywhere we look, there are microbes that assault the product once it is opened. The product starts losing its quality quickly on a microbiological level [7].

## 2. Preservatives and Their Needs

**Figure 1:**
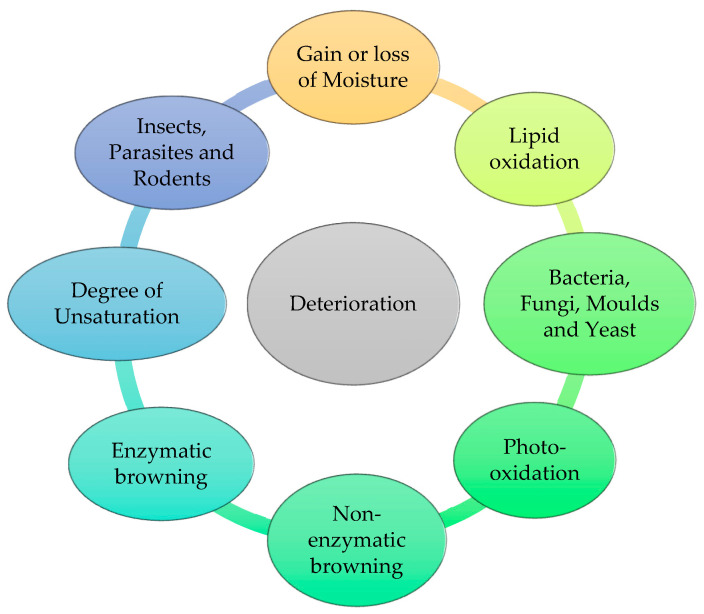
Causes of deterioration of the product.

An antimicrobial preservative is a natural or artificial substance added to non-sterile products in favor of preventing their decomposition due to chemical, enzymatic, or microbial activities or any undesirable chemical changes, most probably caused by microbial growth and lipid oxidation [8,9] (Figure 1). The addition of preservatives is obligatory in the case of products containing water, or organic or inorganic compounds that are at the highest risk of contamination. Products such as creams, solutions, emulsions, suspensions, parental and eye drops, etc. are likely to be perishable, which may further lead to the spoilage of products and result in the loss of some essential properties [10,11]. Therefore, preservatives are meant to be introduced during the manufacturing process to keep the essential qualitative characteristics and organoleptic properties of the product intact by retarding degradation of the product formulation during its shelf life [12]. They keep the product fresher for a longer time so that no undesirable pathogen could attack and bring about unenviable changes in the finished product.

## 3. Factors Monitoring the Effectiveness of Preservatives

**Figure 2:**
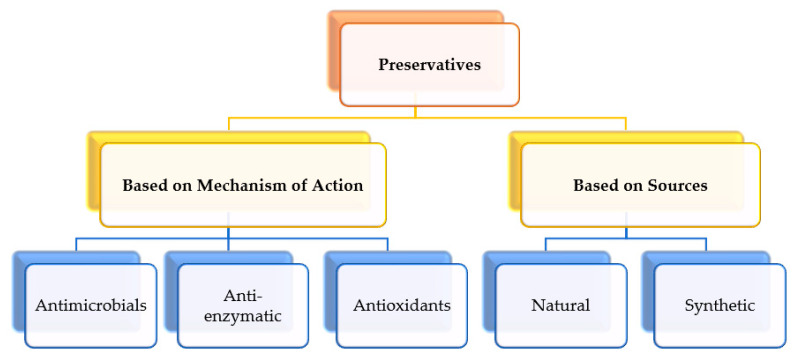
Classification of preservatives.

Every product in the market, whether food, cosmetic, or pharmaceutical, comes with a shelf life. During this period, the product is expected to remain stable retaining its qualitative characteristics, but what if the effect of the preservative is lost? The effectiveness of a preservative depends upon its concentration, solubility, partition coefficient, nature of surfactants used in the formulation having antimicrobial activity, pH, etc. Consumers themselves can easily monitor the loss of effectiveness of a preservative or tarnished product by considering several factors such as the appearance of a greyish-green layer on the top surface of the product, loss of texture, bad taste and odor, change in pH, cloudiness, dryness, bleaching/fading, formation of separation layers, rancidity, staleness, etc. Additionally, some biochemical modifications that affect the organoleptic qualities of foods include hydrolysis, non-enzymatic browning (Maillard reaction), enzymatic browning, lipid oxidation, lipolysis, and proteolysis [13,14,15,16,17]. Therefore, the purpose of adding preservatives is to provide stability and protection against microbial attack, prolong the shelf life, and enhance the efficacy of the product. Before incorporation into miscellaneous products, preservatives have been investigated by researchers worldwide for their decontamination efficacy. The sorting of preservatives attributed to their mechanism of action and sources [18] is depicted below in Figure 2.

## 4. Mechanism of Action of Preservatives

Preservatives, irrespective of their origin and whether natural or synthetic, act through different modes of action such as:

- Cell wall lysis and leakage (phenols and organomercurials).
- Cross linkage (glutaraldehyde).
- Interference with the integrity of the cell membrane (EDTA, quaternary ammonium compounds).
- Inhibition of folic acid synthesis (parabens, benzoic acid, etc.) [19].

## 5. Criteria for the Selection of Preservatives 

As such, there is no single preservative that is effective against all microbial strains and suits all product types. Moreover, manufacturers or regulators are required to use preservatives with the maximum permitted concentration limits specified by the regulatory authorities while taking care of the pH and temperature conditions that may affect the activity. Therefore, the selection of the right preservative is of utmost significance. Several factors based on which one can choose the best preservative are: the purpose of inclusion, proof of efficacy (which in turn depends on the chemical nature, type of interaction, dose, physicochemical properties of a drug, degree of microbial contamination, the influence of pH, storage temperature of the finished product, etc.), broad or narrow spectrum of coverage against pathogens, effectiveness(antimicrobial effectiveness test as specified in different pharmacopeias), safety information, incompatibility interactions, antimicrobial functionality or microbial protection spectrum, dosage form, compliance with a product philosophy, chemical and physical stability, toxicity studies and labeling details of the end product, etc. [20]. 

## 6. Problems Associated with Preservatives

**Figure 3:**
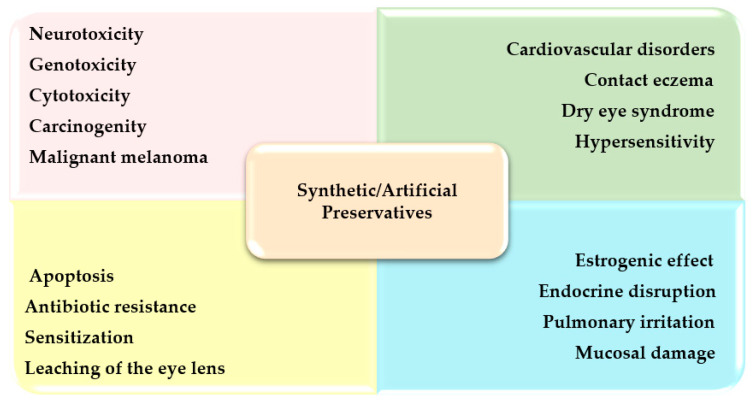
Problems associated with synthetic preservatives.

Nowadays, the use of synthetic/artificial preservatives has become common (as they are cheap and easily available), which has not been widely accepted by consumers as they are aware of the preservatives’ short- and long-term life-threatening adverse effects on health, which is the major area of concern for researchers [21]. The presence of hazardous chemicals in the product sabotages consumer health [22,23]. The various problems/adverse effects associated with the frequent use of synthetic preservatives in various formulations have been summarized in Figure 3. 

Quality control and consumer safety are of utmost importance. Preservatives are therefore necessary to maintain the average life of pharmaceutical preparations, food and cosmetics products but there have also been reports of some adverse effects associated with their frequent use. There exists an immediate need for unwrapping novel and safe preservatives from natural sources for various pharmaceuticals, cosmetics, and food products as it is evident that natural preservatives would be better in terms of the quality, safety, and longevity of the product.

## 7. Preservatives Used in Cosmetics

Cosmetics products are often used by people around the world almost every day and become a part of our lifestyle. Cosmetics products comprise several products ranging from shampoos to deodorants, lotions to creams, foundations to lipsticks, mouthwashes to toothpaste [24,25], and so on, as depicted in Table 1 and Table 2, with the hope of providing protection, maintenance, cleansing, minimizing body odor, beautification and developing an attractive and charming personality [24,26]. They are meant to be frequently applied on external non-sterile parts of the body, especially the skin, and are susceptible to microbial contamination with every use. Cosmetic products containing water, oils, peptides, and carbohydrates offer an ideal environment for the microbial growth of pathogenic organisms, which may further lead to the spoilage of products and result in the loss of some essential properties [10,11]. To prevent microbial growth, undesirable changes, deterioration of the cosmetic product, and chances of skin infection, two distinct classes, namely, antimicrobial and antioxidant preservatives are generally used [24]. Moreover, special measures must be taken by consumers at their end while using cosmetic products to retard their spoilage.

## 8. Food Preservatives

Foodstuffs of various types such as raw food, junk food, fast food, organic food, whole food, processed or unprocessed food, and vegetarian or non-vegetarian food exist across the globe depending upon climatic and geographical conditions. Moreover, foodstuffs are subjected to storage in go downs or houses for handling emergencies [95]. Food products are perishable items with a short shelf life ranging from a few hours to a few days to a few months. For many years, food preservation has been a great challenge for the food industry in terms of ensuring quality, nutritional value, organoleptic properties, and safety [96,97]. Preservatives are generally added to decontaminate the food product and to ensure the stability and safety of the product. The consumer demands chemical-free, fresh, nutritionally rich, tasty, smells good, and ready-to-eat food, which appeals to the taste buds and has a prolonged shelf life too. This has prompted researchers/manufacturers to search and develop natural antimicrobial preservatives. Organic acids and their derivatives depicted in Table 3 are natural compounds that have been exploited by researchers as bio-preservatives for the last few decades. They are found to be inexpensive and effective at decontaminating the food product against the population of food pathogens and are generally recognized as safe (GRAS). Several studies reported that organic acids such as acetic, citric, lactic, propionic, benzoic, and sorbic acids possess an excellent antimicrobial potential, exhibiting a broad spectrum of activity against food pathogens such as bacteria fungi and yeast [98,99,100,101]. 

## 9. Pharmaceutical Preservatives

Pharmaceutical preparations meant to be administered in different dosage forms usually contain additives in addition to the active pharmaceutical ingredient (API). Additives with a certain amount of impurities show interactions with the active drug component, other additives, and packaging materials. Namely, drug–additive interactions, additive–additive interactions, and package–additive interactions, which cause drug degradation [119]. In pharmaceutical solutions, generally known as liquid or oral preparations (solutions, syrups, elixirs, suspensions, etc.), the presence of a higher water content and other ingredients such as solvents and co-solvents in the formulation provides an environment that favors the growth of microorganisms. This microbial contamination causes undesirable chemical alterations in the formulation, which eventually result in product deterioration [120]. 

Non-sterile dosage forms are prone to microbial contamination during the manufacturing process; even sterile dosage forms in multidose containers have access to microbial growth by repeatedly withdrawn doses. Hence, in the pharmaceutical industry, the addition of preservatives becomes necessary to hurdle the prevalence of microbial growth, provide stability, retard deterioration, and increase shelf life. Although preservatives are used in low concentrations, the health issues associated with them cannot be ignored. Some commonly used preservatives in pharmaceutical preparations along with their toxic effects are listed in Table 4. The evaluation of preservative efficacy is completed in conformity with tests prescribed in a pharmacopeia to demonstrate their effectiveness in a product—antimicrobial effectiveness test (USP), efficacy of antimicrobial preservation (EP), and preservation effectiveness test (JP). Based on their chemical nature and structure, preservatives are categorized as: acids (benzoic acid and its salts, sorbic acid), alcohols (benzyl alcohol, chlorobutanol), biguanides (chlorhexidine), esters (parabens), phenols (phenol, m-cresol), mercurials (thiomersal), phenolic antioxidants (BHA, BHT), quaternary ammonium compounds (benzyl alkonium chloride, cetrimide), etc. [121].

## 10. Natural Preservatives and Their Importance

**Figure 4:**
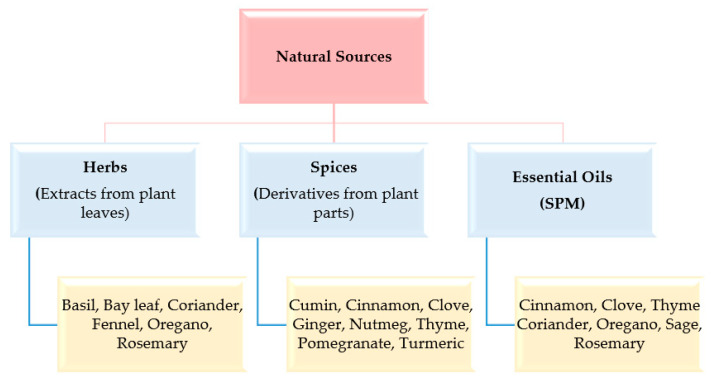
Natural products derived from plants.

Owing to their natural origin, bio-preservatives received lots of attention in recent years as they are much safer and more beneficial when compared to synthetic preservatives. Natural food preservatives are typically of plant, animal, and microbial origin. Natural products, derived from natural plant sources such as herbs, spices, and essential oils, are widely used to give aroma to beverages and mask the disagreeable odor of the constituents in addition to their preservative action (Figure 4) [144]. They are found to increase shelf life naturally by decreasing lipid oxidation. Some of the common conventional methods that can be used to preserve food are drying (natural and artificial), pickling (using salt or vinegar), freezing, high-pressure processing, and the edible coating technique [145].

**Figure 5:**
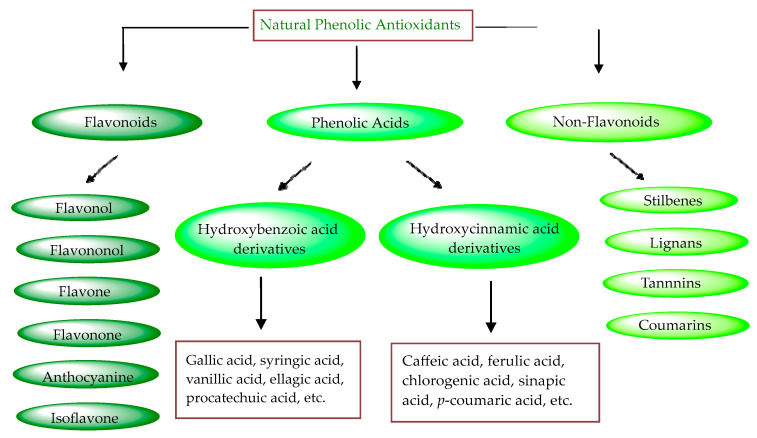
Natural phenolic antioxidants.

Plant phenols are divided into different classes, including simple phenols, phenolic acids, anthocyanins, stilbenes, flavonoids, tannins, lignans, and lignins (Figure 5). Phenolic acids are aromatic secondary plant metabolites that are extensively spread across the plant kingdom. [146]. Currently, there is much scientific interest in their potential protective role against oxidative stress-related diseases. The main edible sources of phenolic acids are fruits, vegetables, cereals, seeds, berries, beverages, olives, and aromatic plants, and they can be found in almost all parts of the plant [147,148]. They occur in the form of esters, glycosides, and amides but rarely in the free form [149]. Despite their origin, these versatile molecules have been reported to possess a wider canvas of biological activities such as antioxidant, antibacterial, antifungal, antiviral, anticancer, anti-inflammatory, anti-diabetic, insecticidal, estrogenic, and keratolytic activities, and many more [150,151,152]. Phenolic compounds are well known for their antioxidant potential and have been extensively used as bio-preservatives to elongate their shelf life, besides their other well-established health benefits. Phenolic acids are non-flavonoid polyphenols that contain a carboxyl group with one or more hydroxyl groups linked to a benzene ring [153,154]. Phenolic acids are known to possess two distinctive carbon frameworks: hydroxybenzoic acid (benzoic acid derivatives) and hydroxycinnamic acid (cinnamic acid derivatives). In these two carbon frameworks, even though the fundamental frame remains the same, the structural variation in the numbers and orientation of hydroxyl groups on the aromatic ring results in a variety of potential derivatives. Hydroxybenzoic acids with seven carbon atoms have a general structure of C6-C1 derived directly from benzoic acid and are present in foods in glucoside form. Some common hydroxybenzoic acids are protocatechuic acid, vanillic acid, syringic acid, and gallic acid. Hydroxycinnamic acids are natural phenylpropanoids having a general structure of C6-C3 derived directly from cinnamic acid and are mostly present in the bound form. Among hydroxycinnamic acids, sinapic acid, caffeic acid, ferulic acid, and *p*-coumaric acid are the most abundant compounds in foods [155,156] (Figure 5). In both these derivatives, the variation lies in their hydroxylation and methylation patterns in aromatic rings.

**Figure 6:**
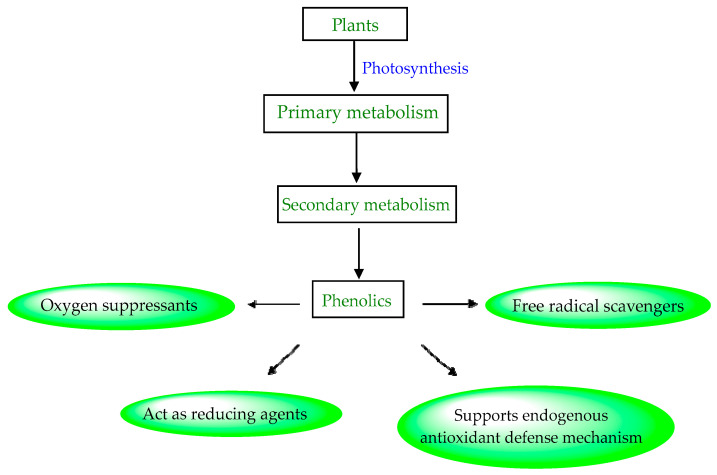
Biosynthesis of phenolic compounds with excellent antioxidant activity.

Structure–activity relationship (SAR) studies of phenolic acids reveal that the reactivity of the phenol moiety imparts antioxidant activity to a greater extent but can also be affected by some other parameters such as the chemical structure, number, position of hydroxyl groups, substituents on the phenolic ring, and esterification of the carboxyl group. They act as reducing agents, hydrogen donors, and oxygen suppressants when added to food products (Figure 6). They also can inhibit the enzymes involved in radical generation and act as free radical scavengers [157,158]. In general, they are used to prolong the shelf life by preventing oxidative rancidity, degradation, discoloration, contamination, and any other undesirable changes from occurring. Because of their presence in natural plant-based foods and their role as dietary antioxidants, along with radical scavenging abilities, they have received a lot of attention from researchers worldwide [146,159].

### 10.1. Natural Plant Constituents as Antimicrobial/Antioxidants

**Figure 7:**
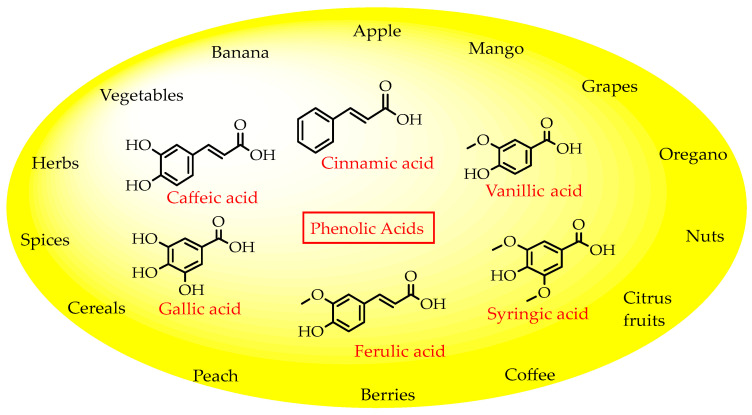
Phenolic acids and their natural sources.

Antimicrobial agents derived from natural sources have been recognized and used anciently in preservation [160]. The importance of natural sources as antimicrobials/antioxidants is well established and this allows them to be selected as potential candidates for novel preservatives (Table 5 and Figure 7).

### 10.2. Applications of Phenolic Antioxidant/Antimicrobials

**Figure 8:**
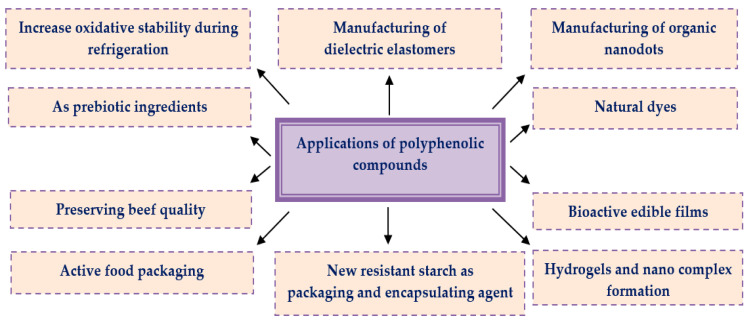
Applications of polyphenolic compounds in different sectors.

Over the last years, naturally occurring fruits, vegetables, herbs, spices, oils, and their extracts attract the attention of researchers as they are regarded as the ultimate sources of phenolic compounds. They have been tremendously utilized/applied by the food, cosmetics, and pharma industries for a better therapeutic efficacy and conservation of substances. Moreover, they are broadly available, reliable, and cheap with minimal toxic effects, which makes them superior to synthetic products in all aspects. Various applications of polyphenolic compounds [188,189,190,191,192,193] in the pharmaceutical, food, and cosmetics sectors have been summarized in Figure 8.

## 11. Permitted/Approved Preservatives

Preservatives are defined as synthetic chemical additives that increase the durability and prolong the shelf life of the product. Although preservatives are effective in preventing a microbial burden in the product, the risk of allergic reactions, sensitization, and other toxicological effects associated with their frequent use cannot be subsided. Preservatives are incorporated in a range of food, cosmetic, and pharmaceutical products, and are an indispensable part of our daily diet and lifestyle as well. Ideally, owing to their necessity in the product, we should either limit their use in the product or opt for natural alternatives, as is the need of the hour. As per European regulations (Directive 95/2/EC), officially recognized and only permitted preservatives have been set down in Annex four of the 7th amendment of the cosmetic directive, which is summarized in Table 6. The Codex Alimentarius Commission (Codex) was established by the World Health Organization (WHO) and the Food and Agriculture Organization of the United Nations to set global standards to ensure fair food trade practices. The Codex General Standard for Food Additives [194] (Codex Stan 192–1995, Revision, 2015) enlisted additional additives, preservatives, and antioxidants, permitted for food that has been thoroughly appraised by an international expert scientific committee, the Joint FAO/WHO Expert Committee on Food Additives (JECFA), to develop specifications on food additives. According to Codex General Standard for Food Additives, a food additive is any substance (preservatives and antioxidants) that has been found acceptable given a green signal for use in foods, such as tertiary butyl hydroquinone (TBHQ), isopropyl citrate, dimethyl decarbonate and ferrous gluconate, etc. In the EU, every additive (preservative) assigned to a code begins with the letter E followed by a three-to-four-digit number. The numbering scheme is under the International Numbering System (INS). Antimicrobial preservatives should be chemically defined and designated by the Chemical Abstract Service Registry Number (RN-CAS) as determined by the Codex Alimentarius Committee [87].

### Amendments in Food Additives

After considering several opinions by the expert committee, FSSAI in its gazette notification adopted an amendment to the Food Safety and Standards (Food Products Standards and Food Additives) 7th Amendment Regulations, 2020, with regard to regulations 3.1 dated 16 September 2020, which came into force by 1 July 2021 [195,196]. Under Food Category System 6.2 and 7.2.1 as per the new regulations, the following changes have been introduced as given below in Table 7.

After the consideration of the Scientific Opinion on the re-evaluation of sorbates as food additives/preservatives, the European regulations finally decided to adopt an amendment in Annexes II and III to Regulation (EC) No 1333/2008 and Annex I to Regulation (EU) No 231/2012 [197,198], by excluding calcium sorbate from the list of officially permitted food additives with the backdrop of lack of appropriate genotoxicity data, based on which calcium sorbate can no longer enjoy the status of being an authorized food additive.

Climbazole (CAS 38083-17-9) is a cosmetic preservative that has been used in various cosmetic preparations such as face creams, lotions, foot cream, rinse-off shampoos, and many others, at an allowed concentration of 0.5%. Experts from the Scientific Committee on Consumer Safety (SCCS) found that the use of climbazole at 0.5% in cosmetic products is not safe and may pose serious health issues to consumers (SCCS/1506/13, 2017). To ensure consumers’ safety, the European Commission amended the cosmetic regulations and decided to impose restrictions on the use of climbazole as a preservative in cosmetic products. Considering the harmful effects associated with the use of climbazole, Europe’s Scientific Committee on Consumer Safety (SCCS), SCCS/1600/18, set new regulatory limits to climbazole for its safe use in cosmetic preparations [199,200]. This regulation took effect on 22 May 2019, while amendments to Annex V were applied from 27 November 2019. Currently, as per the new regulatory limits, the allowed maximum concentration has dropped down to 0.2% in face creams, hair lotions, and foot care products and 0.5% for rinse-off shampoo (Table 8). These restrictions were implemented for all new products as well as the products already available in the EU market containing climbazole.

Hydroxyethoxyphenyl butanone (HEPB, CAS 569646-79-3), also known as ethyl zingerone, is intended to be used as a cosmetic preservative and has been a new entry to the family of authorized preservatives in cosmetic products [89]. In 2019, based on safety assessment studies, the SCCS recommends the inclusion or entry of HEPB in the list of approved preservatives. On 6 November 2019, HEPB was officially enlisted in Annex V of the European Cosmetic Regulation 1223/2009 [201] with the recommended safe maximum limit concentration of 0.7% *w*/*v* in all cosmetic products concerning eye irritation [200]. 

## 12. Unfair Practices in Trade

For holding a good market position globally, neck-to-neck competition can be seen among the brands and companies advertising their products. For ages advertising has been in practice, and companies spend a handsome amount of money just to expand their business globally by persuading consumers to buy the product [202]. Advertising through different mediums such as social media, print media, digital media, etc. provides a wider canvas to overhype a particular brand or product. Although we are progressing technologically very fast, we end up in a world where spurious realities are created by the media. Nowadays, advertising is one of the effective mediums of communication between manufacturers and consumers. It is a way to attract, connect, and capture the consumers at the other end with undeniable influence on their mindset, but becomes problematic if they are misleading in any way [203].

**Figure 9:**
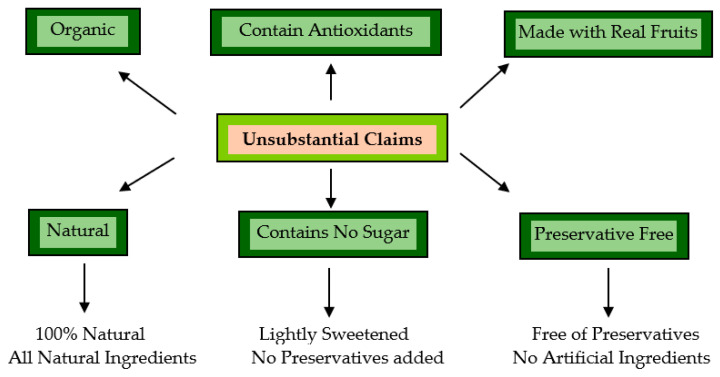
Some commonly used exaggerated claims.

Today, advertising has become a necessary tool for producers to burgeon their business and for consumers to increase their awareness, to select the best out of the box [204]. Advertising is an act of selling the concept “what you see is sold” playing out with the consumer sentiments. The advertising industry is dodgy, indulged in unethical practices by running false campaigns for peddling an average product, poaching the common man with illusory wordplay [205]. Common men are vulnerable to advertising and are unceasingly bombarded with pseudo realities manufactured by very sophisticated mechanisms. Moreover, there are different means of the product packaging systems available in the market to ensure their safety. These packaging systems act as a barrier between the product and consumer as consumers do not have direct access to the facts of the product. In such circumstances, product labels are the only means of providing information about the hidden product. Before we discuss the technicalities of the matter, one must understand the meaning of “claim”. As per the Joint FAO/WHO Codex Alimentarius Commission, 1992, this claim is referred to as a message in any form that states or implies that a food has particular characteristics with regards to its quality, nutritive value, quantity, health functional food, etc. [206]. Today, many companies have adopted unethical/illegal practices to prop up their false claims to mislead consumers and to make large profits at the cost of their health, and these are under the lens [203]. Therefore, every nation appoints government officials to design and adopt policies comprised of laws, regulations, and guidelines that products need to comply with. In 2015, under the legislative framework, the Government of India appointed a self-regulatory body, the Advertising Standards Council of India (ASCI) arm that twists advertising agencies and coerces them to follow laws and regulations. ASCI analyzes and scrutinizes the advertising content and entertains the complaints against the advertisement, whether the advertisement builds any deceptive or exaggerated claim that may mislead the consumer in any way. It is a commitment to healthy and fair competition. In recent years, consumers have now become more aware of the toxic effects of synthetic chemicals on their health, therefore, they are inclined towards traditional natural alternatives being incorporated in the products. To comply with the elevating demand for natural alternatives, companies adopted tricky advertising strategies (Figure 9) to market their products to make an unfair profit [207]. An advertisement and label become deceptive or misleading if they bear a bogus claim as a part of a marketing strategy to advertise the product:

Other false claims include—hiding or misstating the number of ingredients, labeling claims of extraordinary health benefits, highlighting or overstating the nutrient value, using exaggerated claims such as best/perfect, delicious, etc.

## 13. Misleading Labeling and Advertising

Advertising may influence the thought process of the consumer both positively and negatively as well. The content is deemed to be negative, unethical, and unfair if it misleads consumers and represents an illusory picture of the product, making them believe that a particular product is superior to other rival products available in the market to promote the sale. Some unfair or deceptive practices based on exaggerated claims [205,208,209] are summarized as follows:

- Falsely representing food with a name similar to the prescribed name of an oriental medicine so that consumers misperceive the food as a drug.
- Incorrect representation of food with a mark to depict the food as a healthy functional food.
- Labeling or advertising products as “Preservatives not added” and “Free of preservatives”.
- Falsely representing names other than those defined under the Standards and Specifications for Food Additives announced by the Ministry of Foods and Drug Safety.
- Purposely representing that goods are of a particular grade, quality, and standard.
- Labeling or advertising 100% when the product contains ingredients other than the raw material specified on the label. For instance, 100% orange juice (containing citric acid and other acidity regulators).
- Labeling or advertising food with effects or properties that it does not possess.
- Labeling or advertising food bearing special characteristics regarding nutrient content.

As per European regulations on misleading advertising in Directive 2006/114/EC [210], misleading advertisements are deceptive based on unsubstantial and exaggerated claims that represent a false picture of the product and violate the customer’s right to know about the products they are purchasing. The sale of such a misbranded product is against the law. Misleading advertisements are a serious ill issue for society that needs to be addressed. Therefore, the advertising industry requires the following laws and regulations to ensure the credibility and authenticity of the common man [211,212].

## 14. Recent Trends in Preservation

Scores of preservatives, whether natural/synthetic, are used in various products available in the market. A clear trend towards mild substances can be seen, particularly organic acid-based preservatives are quite popular nowadays. Preservatives are generally incorporated to ensure the safety of the product but can deteriorate the product if used inadequately. Based on several reports on the risk of sensitization associated with the use of preservatives, the Scientific Committee on Consumer Products in Europe and the Cosmetic Ingredient Review in North America introduced changes in legislation to pose restrictions on their maximum permitted concentrations or even banned them in some cases [48,213]. Companies are now looking for new natural alternatives with the potential to live up to the expectations of both producers and consumers.

### 14.1. Controversial Preservatives

#### 14.1.1. Parabens

Based on early assessments, parabens are classified among the oldest and most commonly used preservatives in various food, cosmetics, and pharmaceutical products. Parabens and their esters are generally used in topical formulations as they are chemically stable and have broad-spectrum antimicrobial activity [214,215]. In recent years, as far as safety is concerned, parabens have been the most criticized among other preservatives. Parabens are accused of causing various health issues such as skin sensitization, allergic dermatitis, endocrine disruption [216], estrogenic effects, interference with male reproductive functions, breast cancer [46], the development of malignant melanoma, gestational diabetes mellitus, etc. [217,218], and their usage in products will be questioned remain a long-lasting challenge. Darbre et al., identified the presence of different paraben esters in human breast tumor tissue samples [46]. Keeping in mind the adverse effects, consumer fear, market trends, and the inclination of consumers towards organic products, manufacturers were prompted to replace paraben or lower its limit concentration in their products [219]. Even some manufacturers set a new trend to write “Paraben-free” on the product label that flourishes in the market everywhere. It is noteworthy that the use of “preservative free” on the labels is EU compliant while “paraben free” is not. For the sake of the safety of both the product and the consumer, the use of preservatives has been subjected to certain restrictions [215]. After the thorough evaluation of parabens based on a cumulative study by the Scientific Committee on Consumer Safety (SCCS) of the European Commission, the use of parabens was restricted to lower concentration limits [220]. Based on pieces of evidence related to the toxicity of parabens, the FDA approved new limits for the use of parabens, i.e., 0.1% limit concentration in foods, 0.1% for solo, and 0.3% limit concentration for a blend of the compound in pharmaceuticals [221]. In cosmetics, the concentration limit was reduced from 0.4% to 0.14% and was prohibited in the case of children’s products [215,222,223]. In countries such as Denmark, the use of parabens is illegal and deemed to be unsafe, therefore, they were banned, but in other countries including India, the use of parabens is still legal [218,224]. In cosmetics, owing to their natural origin, parabens are preferentially replaced by phenolic compounds as they are gentle, non-irritating, and do not release formaldehyde.

#### 14.1.2. Phenoxyethanol

Phenoxyethanol (122-99-6) is an ether and aromatic alcohol with a large antimicrobial spectrum against various bacterial strains and yeast. According to European Scientific Committee on Consumer Safety Cosmetics Regulation (EC 1223/2009) [222], phenoxyethanol is authorized as a safe preservative for all consumers including children when used at a maximum concentration of 1%. Nowadays, parabens have been largely replaced by phenoxyethanol in many cosmetic formulations [225]. Keeping in mind the elevating demand for natural ingredients by the consumer, the globally recognized cosmetic brand manufacturers took the criticism seriously and introduced synergistic blends of multi-functional natural ingredients in their products. They are all set to go completely paraben-free across the full product range shortly.

#### 14.1.3. Formaldehyde Donors

Considering the allergic skin reactions caused by using of formaldehyde and formaldehyde-releasing preservatives (DMDM, hydantoin, imidazolidinyl urea, diazolidinyl urea, sodium hydroxymethyl glycinate, and quaternium-15) in various personal care products, the EU and GSO restricted their concentration limit to 0.2% *w*/*w* in all cosmetics products and 0.1% *w*/*w* for oral hygiene products. According to Annex VI of the Cosmetics Directive 76/768 EC and Gulf Technical Regulation for Safety Requirements of Cosmetics and Personal Care Products (GSO 1943/2016) [226], final products must enumerate with a word warning “contains formaldehyde” on their label containing formaldehyde or its releasers in the concentration exceeding the maximum allowable limit, i.e., 0.05% *w*/*w* [227]. 

### 14.2. Natural Preservatives

In recent years, the market trend has changed dramatically as per the consumer’s perception and acceptance of natural alternatives over synthetic ones. Consumers are interested in fresh and natural products with no added preservatives [228]. Demands have been pouring in from all quarters for natural, herbal, and organic products. In recent decades, manufacturers have adopted different efficacious methods of extending shelf life during commercialization and have gained relevance. In this context, bio-preservatives received considerable interest and have been exploited for their antimicrobial potential by researchers worldwide to extend their shelf life.

### 14.3. Nanotechnology in Food Preservation

In recent years, nanotechnology is the most active and fastest growing active research field with the unique ability to manipulate matter in nano dimensions and impart novel properties to the materials. Nanotechnology has numerous applications in various fields, but its role in food preservation is remarkable. Nanotechnology deals with the synthesis of various nanomaterials of different dimensions such as nanoparticles, quantum dots, nanorods, nanotubes, nanocapsules, nanoemulsions, etc. [229,230]. Owing to their physicochemical nature and antimicrobial potential, nanomaterials can offer a solution in food packaging and preservation [231,232]. In functional foods, bioactive components such as carbohydrates, proteins, and vitamins, etc. are susceptible to microbial deterioration, which ultimately leads to the degradation of food. Nanoencapsulation protects the biologically active components by providing a protective barrier against moisture and gas exchange that enables the retarding of their chemical degradation, provides stability, and facilitates their controlled release. The nanoparticles are responsible for developing mechanical and heat-resistant properties that eventually prolong the shelf life [233]. Food ingredients are prone to degradation during processing and oxidative deterioration during storage, therefore, efficient packaging systems are required to overcome this problem. When we talk about an efficient packaging system, the packing material is of paramount significance for maintaining the quality of foodstuffs. The packaging concept has been introduced to serve important functions such as product containment, presentation and convenience, preservation, quality protection, and to provide storage history of the concerned product. Food packaging provides a protective barrier to fresh fruits and vegetables against air, light, moisture, dryness, etc., thus playing a critical role in the preservation of foodstuffs. Nano packaging includes “active”, “smart”, and intelligent food packaging systems [234] such as biodegradable nanocomposites, nanoclays, and nano edible coatings on the food surface with excellent mechanical strength and barrier properties that effectively improve food durability and contribute to the shelf life of the food product. Intelligent food packaging with nano sensors to monitor the food for pathogen detection and alert consumers to the safety status of food are now in practice [235]. Edible coatings and films on the food surface are meant to be consumed with packaged food. It becomes necessary that the ingredients used in the packaging should be classified as GRAS. The applications of various nanoparticles (NPs) and nanomaterials have been briefly summarized in Table 9.

### 14.4. Preservation without Preservatives

When natural food is processed, by using any means, some chemicals are added, which ultimately results in the loss of its nutritional value. The processed food needs to be consumed within a limited time as many additives were added during its processing, otherwise, the food will begin to deteriorate. The deteriorated food affects the color, taste, appearance, smell, stability, and shelf life of foods badly and diminishes their nutritional value and quality characteristics [271]. To protect these properties, several preservatives are commonly used to keep the food fresh and safe from spoiling for a longer period. The inclination of consumers toward natural additives is increasing gradually [272]. Currently, researchers are left with no other option and have been rigorously looking for incorporating different additives derived from natural sources in the products. Today, we can easily find products in the market with a label indicating “No Added Preservatives” or “100% Natural”. Well, it is not always necessary to add preservatives to the finished product. There are some conditions where preservatives are not required to be added anymore, such as when there is a lack of water, when the ingredients present in the formulation are self-preserving due to their antimicrobial capabilities, when preparations were created in a single batch and are supposed to be consumed immediately in no time, and when the pH of the medium is such that it will not stand the microbial population easily, i.e., either <3 or >9.

An alternative strategy to be exercised when preservatives are indispensable in pharmaceutical preparations is to prepare a single dose and use it instantly or devise a limited amount meant to be consumed within a short period. This way, the use of preservatives in pharmaceutical preparations can be contraindicated [273]. Although fruit juices of different brands with added preservatives are available in the market, it is always better to consume freshly prepared fruit juices to avail the maximum benefits. Otherwise, carbohydrates present in the juices lead to the formation of Maillard browning products [274,275]. The premature spoilage of cosmetics products can be minimized by disinfecting the working space and containers properly, making products in small batches, storing them in a refrigerator at low temperatures, using a spatula (instead of a finger) to use the product, avoiding exposure to moisture and sunlight, etc.

### 14.5. Self-Preserving System

Microbial contamination is one of the major inevitable issues that needs to be addressed. Preservatives are often added to control microbial bioburden, i.e., prevent product deterioration by inhibiting microbial proliferation and sufficiently extending the shelf life of the product. The use of preservatives is found to be associated with many toxicological effects; therefore, it is preferred to add preservatives at concentration levels as low as possible. However, the inadequate level of preservatives is again of no use as it gives room to microbial contamination and makes the product susceptible to deterioration. To achieve preservation without adding preservatives, reliable and effective methods for extending the shelf life must be considered, such as the use of bio-preservatives that offer better packaging formats [276]. An alternative approach to achieve the balance between the antimicrobial efficacy and toxicological effects of antimicrobial preservatives is to include multifunctional components in the formulation, which enhance the overall antimicrobial activity of the formulation in addition to their primary function. Self-preserving systems or formulations come into the picture, in which the traditional single chemical preservatives have been replaced by other ingredients of the formulation with strong antimicrobial properties to combat microbial growth [277]. In cosmetic products, many cosmetic ingredients other than preservatives, such as alcohol, antioxidants, biomimetic phospholipids, chelating agents, fatty acids, essential oil, and surfactants, are found to possess antimicrobial properties. Ophthalmic formulations, which are generally formulated as isotonic buffered solutions, also include multifunctional ingredients in the composition to enhance the overall antimicrobial activity of the formulation. However, these multifunctional ingredients have not yet been recognized as preservatives by European regulations and are not listed in annex four of the 7th amendment of the Cosmetic Directive among officially permitted preservatives [278,279]. The production of self-preserving systems or formulations is based on the principles of “Hurdle technology” which involves the intelligent and careful combination of multifunctional ingredients, with mild individual impact, to minimize or eliminate the use of toxic chemical preservatives [280]. The principles of self-preserving technology involve a synergistic combination of preserving factors or hurdles to keep a constant check on the microbial populations and offer high quality and safety. The stringent maintenance of aseptic conditions through the strict adherence to GMP requirements, airless packing to prevent the introduction of outside microorganisms to improve preservation, and water activity is decreased to discourage the microbial growth, bio-preservatives, and pH control to decrease or minimize the proliferation rate of microbes and multipurpose antibacterial components [281]. Below are listed some multifunctional ingredients with antimicrobial properties (Table 10) [279]: 

### 14.6. Pulse Electric Field (PEF)

In recent years, the non-thermal processing technique, pulse electric field (PEF) emerged as an effective and potential tool in the field of food preservation and has gained popularity over conventional thermal methods of food preservation. Studies revealed that this technology enables the inactivation of microorganisms causing spoilage of the food product [282]. Wouters et al. reported that this food preservation technology involves the application of short high-voltage pulses of electricity with a short duration ranging from micro to milliseconds, generating an electric field resulting in microbial inactivation of the product (fruits, vegetables, juice, and dairy products) placed between the electrodes at a low temperature, which disrupts the cell membrane matrix of the food, thereby extending its shelf life [283]. PEF technology has a wide range of applications in various food products such as watermelon [284], blueberries [285], apple juice [286,287], red beet [288], olive paste [289], and clover sprout [290]. This technique aims to provide high-quality food to the consumer with minimal detrimental influence on the food quality. This technology is superior in keeping the sensory, nutritional, and physical attributes of the food intact [291]. Owing to its low temperature and short treatment time, the PEF technique exhibits immense potential for shelf-life extension in addition to its microbial decontamination properties [292].

## 15. Legislation for Food, Cosmetics, and Pharmaceutical Products around the Globe

**Figure 10:**
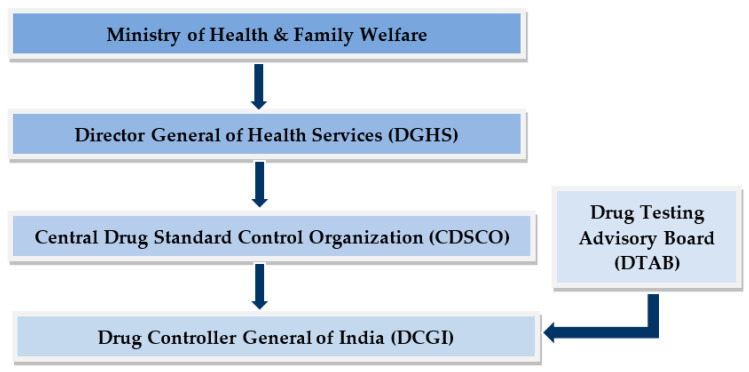
Flowchart of organizational structure of Indian pharma regulatory system (CDSCO).

Every nation needs effective legislation to regulate and control the formulation of various products by imposing laws and setting up guidelines for ensuring the safety of both products and consumers as well (Figure 10). Based on the safety evaluations, some restriction limits and conditions are legally being imposed by regulatory bodies or agencies throughout the world for the use of additives in the product. It is legally required that preservatives must be used in compliance with the regulations specified in the legislation to certify the safety of the finished product. Several ICH guidelines should be taken into account for the inclusion of antioxidant and antimicrobial preservatives in the product. In the EU, every additive (preservative) assigned to a code begins with the letter E followed by a three-to-four-digit number. The numbering scheme is by the International Numbering System (INS). Antimicrobial preservatives should be chemically defined and designated by the Chemical Abstract Service Registry Number (RN-CAS) as determined by the Codex Alimentarius Committee [87].

### 15.1. Cosmetic Legislation

Cosmetic goods have a large global market around the world. Effective legislation is required to regulate the market, trade, quality of products, and safety of the user. Therefore, every nation has legislation run by regulatory authorities, having its own rules and regulations, to regulate the overall activities. Instead of understanding the regulatory aspects in different countries, we need to understand how these regulatory bodies function [293]. Among the recommended regulations worldwide, the legislation of the USA, Europe, and India receives considerable interest and their various regulatory aspects are summarized in Table 11. In the US FDA, as per the FD&C Act, a product may be regarded either as a drug, cosmetic, or (in contrast to the position in the EU) as both a drug and a cosmetic. Both the FDA and EU declare that cosmetics need not be sterile and contain specified microorganisms within restricted limits, which may not influence the product characteristics [294,295,296]. Council Directive 1223/2009 EC of Europe listed prohibited substances [297], which cannot be a part of the formulation of a cosmetic product, in Annex II, while regulated substances in which restriction limits are set for certain allowed substances, especially preservatives, are included in Annex VI. In India, cosmetic goods are regulated under the Drugs and Cosmetics Act, 1940, and the Drugs and Cosmetics Rules of 1945, in association with the Bureau of Indian Standards (BIS) to set the standards.

### 15.2. Food Legislation

Compelling food grades and governing systems are mandatory to unify quality in every facet of food production and service to encourage the production of safe and healthier food, aid advancement in international trade, and prohibit the sale of deteriorated or unsafe/fraudulent food. Therefore, the legislation needs to be updated from time to time (Table 12). Food standards are regulated at different levels such as company standards, national standards, regional standards, and international standards (Codex Alimentarius Commission (CAC), International Organization for Standardization (IOS), and World Trade Organization (WTO)). Food Product Standards and Food Additives (FSS) Regulations 2011 defines “preservative” as a substance capable of retarding the deterioration process of the food item. The careful implementation of laws formed by regulatory bodies reassures impartial trade practices through adherence to the basic provisions of the food law, which avoids unfair competition. The EU defines different categories of food additives and sets a list of authorized food additives and their condition of use. The Codex Alimentarius Commission (CAC) provides a collection of various food safety standards adopted internationally. The CAC has become the single international platform, officially recognized by WTO, for different countries to develop national standards. The CAC mainly focuses on ensuring fair food trade practices and consumer health protection. Legislative bodies restrict the number of additives/preservatives to the lowest possible level allowed to be incorporated in the food product to execute its yearning effect. The chemical preservative is generally recognized as safe (GRAS) if it is covered by food additive regulations, prescribing conditions of safe use [306,307].

### 15.3. Pharmaceutical Legislation

Earlier the additives were assumed to be a passive part of the formulation. Based on several reports, these assumptions were put to an end when a chemical group of additives was found to interact with other ingredients of the formulation showing toxic effects. Preservatives form an indispensable part of the formulation; they have to be used in the right proportion so that they do not dominate the pharmacological action of other active ingredients. National pharmacopeias depict quality prerequisites for pharmaceutical additives. Pharmaceutical regulations comprise legal, administrative, and technical measures to ensure the safety and efficacy of the drug. To discharge their duties effectively, regulatory authorities put a great emphasis on the manufacturing of pharmaceutical products to maintain high standards without compromising the quality, efficacy, purity, and safety of the finished product at every step. The objective of drug regulatory legislation is to lay down standards for regulating pharmaceutical products through the establishment of a drug regulatory authority. The International Conference on Harmonization (ICH) provides a platform that gathers the regulatory authorities of different countries (Table 13) and experts from the pharmaceutical industry to analyze various aspects of product registration. The ICH issues guidelines on the technical requirements for drug products containing new ingredients and is subjected to revision from time to time to harmonize with the lines of the WHO and US FDA protocols for maintaining regulatory obligations to protect public health [316]. 

## 16. Conclusions

It is now evident that the consumption of natural foods containing a good amount of phenolic compounds proves to be beneficial for human health as they help in minimizing the risk of some serious ailments. Polyphenolic compounds are excellent antioxidants and bio-preservatives, which made the researchers curious to explore their immense bioactive potential and are found to be useful in a wide variety of applications. Synthetic preservatives are also able to sufficiently extend the shelf life of the perishable product, although they have been reported to have many side effects with harmful impacts on our health. Keeping this in mind, we encourage the use of preservatives made from natural plant products rather than synthetic ones. As far as food items are concerned, it is always better to consume fresh food free of preservatives, if at all possible. In an attempt to rugby-tackle illegal or deceptive practices based on unsubstantial and exaggerated claims that represent a false picture of the products, regulatory agencies posed restriction limits on the use of additives around the globe to take control over these outrageous misleading advertisements and protect the common man from being duped.

## Figures and Tables

**Table 1:** Different classes of cosmetics and personal care products.

Class	Products
Leave-on products	Deodorants
	Antiperspirants
	Body lotion
	Face/Hand cream
Rinse-off products	Shampoo/Hair Conditioner
	Shower gel
Make-up/Embellishment products	Liquid Foundation
	Eyeliner/Mascara
	Lipstick
	Perfumes
Oral care products	Toothpaste
	Mouthwash
Protective products	Anti-wrinkle creams
Corrective products	Beauty masks
	Hair dyes
Maintenance products	Shaving/Moisturizing creams
Active products	Antiseptics

**Table 2:** List of existing cosmetic preservatives and their problems.

Preservative and Its Chemical Structure	Category	Toxic Effect	Cosmetics/Personal Care Products	References
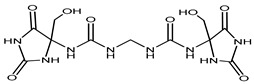 Imidazolidinyl urea	Formaldehyde releaser	Carcinogenic	Eyeliners, mascara, eye shadows, foundation, shampoos, sunscreens, powders, tanning creams, make-up removers, concealers, moisturizing lotions, etc.	[27,28]
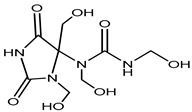 Diazolidinyl Urea	Formaldehyde releaser	Carcinogenic	Blush, eyeliners, eye shadows, lipsticks, foundation, face powders, concealers, moisturizers, hand wash, sunscreens, hair colorants, etc.	[28]
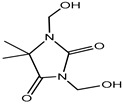 DMDM Hydantoin (Glydant)	Formaldehyde releaser	Carcinogenic, dermatitis	Exfoliants, shampoo, hair smoothing products, liquid soaps, creams, lotions, nail polish, nail glue, etc.	[29,30]
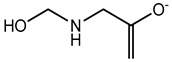 Sodiumhydroxymethyl glycinate (SHMG)	Formaldehyde releaser	Carcinogenic, skin irritant	Body sprays, baby wipes, cleaning agents, shampoo, soaps, hair conditioner, etc.	[28,31]
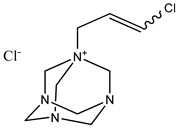 Quaternium-15	Formaldehyde releaser	Sensitization	Blush, bronzer, eye shadow, foundation, baby products (baby oil, shampoo, wash bath), shaving creams and gels, sunscreens, etc.	[28,32,33]
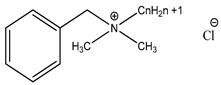 Benzalkonium chloride solution I.P (BAK)	Quaternary ammonium compound	Cytotoxicity, dry eye syndrome, pulmonary irritation, nasal mucosal damage, bacterial resistance, genotoxicity, apoptosis, leaching of eye lens, etc.	Deodorants and body spray, baby wipes, moisturizers, body washes, cleansers, etc.	[34,35,36,37,38,39,40,41,42]
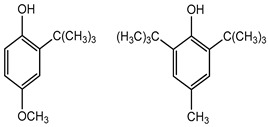 BHA BHT	Phenolic compounds	Endocrine disruptors, neurological problems, metabolic dysfunction, behavioral issues and may cause cancer	Lipstick, lip glosses, moisturizers, lotions, sunscreen, fragrance, clean and clear foaming face wash, face wash, soap, shampoo, deodorants, etc.	[28,43]
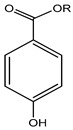 R = an alkyl group Methylparaben, Ethylparaben, Propylparaben, Isobutylparaben, Butylparaben	Paraben	Estrogenic effect and interfere with male reproductive functions, breast cancer, contact eczema, development of malignant melanoma, etc.	Creams, face powder, blusher, face pack, face scrub, nature’s facial kit, face wash, etc.	[28,43,44,45,46,47,48]
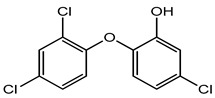 Triclosan and triclocarbans	Antimicrobial agent	Antibiotic resistance in bacteria, endocrine disruptor, neurotoxicity, skin sensitization, reproductive and developmental toxicity, genotoxicity, phototoxicity, carcinogenicity etc.	Foundation, lip gloss, mascara, body spray, perfume spray, medicated soaps, toothpaste, deodorants, hand wash, face wash, mouthwash, etc.	[28,49,50]
Kathon CG: 3: 1 mixture of 5-chloro-2-methyl-4-isothiazoline-2-1 and 2-methyl-4-isothiazolin-2-one	Isothiazolinones	Allergic contact dermatitis	Body washes, liquid soaps, conditioners, shampoos, wipes, etc.	[51,52]
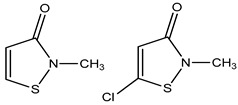 Methylisothiazolinone, Methylchloroisothiazolinones	Isothiazolinones	Skin irritant	Shampoos, conditioner, liquid hand wash, soaps, face wash, etc.	[48,53,54,55,56]
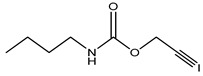 Iodopropynylbutyl carbamate (IPBC)	Belongs to the carbamate family of biocides	Sensitization	Lip balm, lotions, hair dye, hair colorants, etc.	[32,52]
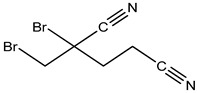 Methyldibromoglutaronitrile (MDBGN)		Contact allergy	Body lotion, hand lotion, facial lotion, sunscreen lotion, shower gels, etc.	[32,52]
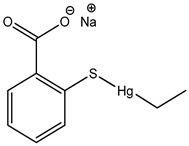 Thiomersal	Mercurial	Kawasaki’s Disease, genotoxicity, apoptosis, cytotoxicity, neurotoxicity, neurodevelopmental and CVS disorders	Eye make-up products	[57,58,59,60,61,62,63,64,65]
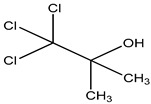 Chlorobutanol	Alcohol	Not used in aerosol dispensers, cytotoxicity, retinal toxic, irritation	Face wash, creams mouthwashes, ointments, etc.	[66,67,68]
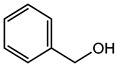 Benzyl Alcohol	Aromatic alcohol	Contact dermatitis, mucous membrane irritation, CNS depression lethargy, metabolic acidosis, toxicity on fatal development and respiratory abnormalities, etc.	Creams, baby lotion, sunscreen SPF 50, baby wipes, etc.	[69]
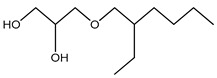 Ethylhexylglycerin	Glyceryl ether	Contact allergen and impairs membrane integrity, etc.	Baby cream, shower gel, moisturizing lotions, skin peels, face masks, body butter, shampoos, conditioner, etc.	[70,71,72]
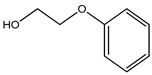 Phenoxyethanol	Glycol ether	Allergic and skin irritation	Hand sanitizer, perfume, F-foundation, blush, lipstick, mascara, eye shadow, hand cream, hair color and spray, lip balm, nail polish, baby wipes, etc.	[73,74,75,76]
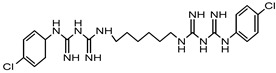 Chlorhexidine	Amidines	Ototoxic, nausea, stomach irritation, respiratory distress syndrome	Conditioner, creams, toothpaste, deodorants, antiperspirants, etc.	[77,78,79]
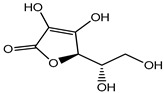 Ascorbic acid	Organic acid	Skin irritation	Anti-aging creams and skin toners, etc.	[80,81,82]
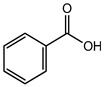 Benzoic acid	Organic acid	Carcinogenic, irritation, metabolic acidosis, asthma, convulsions, etc.	Face wash, shampoo, body wash creams, etc.	[83,84,85]
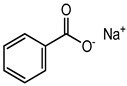 Sodium benzoate	Organic acid	Carcinogenic, irritation, metabolic acidosis, asthma, convulsions, etc.	Body cleanser, toothpaste, etc.	[83,84,85]
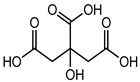 Citric acid	Organic acid (Alpha-hydroxy acid)	Sensitization and irritation, etc.	Facial kit, face wash, serum, scrubbers, baby facial toners, exfoliating, cleaning products, etc.	[86]
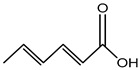 Sorbic acid	Organic acid (Monocarboxylic acid)	Itching and irritation, etc.	Body lotion, eye cream, anti-aging face serum, eyeshadow, cleansing wipes, bronze powder, skincare products, etc.	[87]
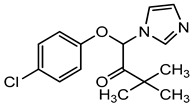 Climbazole	Antifungal agent	Skin irritation includes redness, rashes, and itching	Hair lotions, face creams, foot care products, and rinse-off shampoo, etc.	[88]
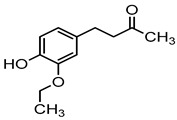 Hydroxyethoxyphenyl butanone		Slight ocular irritation	Rinse off, oral care, and leave-on cosmetic products	[89,90]
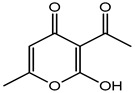 Dehyderoacetic acid (DHA)	Organic acid	Minimal eye irritation, allergic contact dermatitis	Shampoo, lotions, creams, mousses, sunscreens, baby care products, oral care products, foundations, powders, wipes, etc.	[91,92]
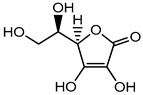 Erythorbic acid	Stereoisomer of ascorbic acid	Sensitization	Hair and nail products	[93]
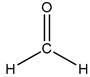 Formaldehyde	Naturally occurring organic compound	Contact dermatitis, irritation and leukemia on prolonged exposure, etc.	Nail polishes, hair gels, soaps, lotions, makeup products, shampoos, etc.	[94]

**Table 3:** List of existing food preservatives and their problems.

Preservatives & Chemical Structure	Toxic Effect	Food Products	References
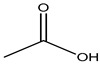 Acetic acid	Breathing difficulty, swelling, irritation	Preserved fish, preserved meat, cooking oil, curry powder, processed cheese, instant puddings, butter, bread, mozzarella cheese, baby food, etc.	[102,103]
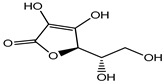 Ascorbic acid (Vitamin C)	Nausea, vomiting, headache, heartburn and cramps	Fruit juices, jams, beer, soft drinks, cider, cereals, canned tuna, glaze for frozen fish, etc.	[81]
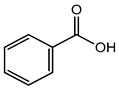 Benzoic acid	Carcinogenic, irritation, metabolic acidosis, asthma, convulsions, etc.	Fruit juices, soft drinks, coffee extracts, pickles; pineapple marmalade with pectin; relishes; tomato paste; tomato pulp; tomato puree, etc.	[83,84,85]
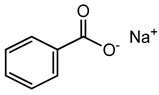 Sodium benzoate	Allergy, asthma, skin rashes, hyperactivity, liver cirrhosis, Parkinson’s disease, etc.	Chayavanprash, fruit juices, fruit cocktails, tomato ketchup, pasta sauce, milk products, jams, soft drinks, salad dressings, pickles, margarine, etc.	[83,84,85,87]
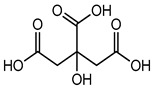 Citric acid	Numbness, rapid weight gain, cramps, mood changes, severe stomach pain, diarrhea and convulsions, etc.	Fruit juices, soft drinks, meat, frozen food, chips, biscuits, ice creams, soda, wine, etc.	[86]
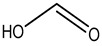 Formic acid	Nausea, vomiting, dizziness, headache, skin allergy etc.	Livestock feed	[104]
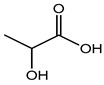 Lactic acid	Brain fog, gas, and bloating	Yoghurt, olives, cucumber pickles, salad dressing, cheese, frozen desserts, soda, etc.	[105]
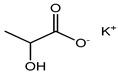 Potassium lactate	Brain fog, gas, and bloating	Meat and poultry products	[105,106,107,108]
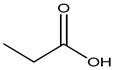 Propionic acid	Corrosive, increase resistance to glucagon, norepinephrine, and insulin	Bakery products, gelatin, milk, and milk products such as cheese, yoghurt, etc.	[87,109]
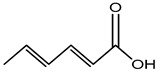 Sorbic acid	Contact dermatitis and urticarial, etc.	Fruit juice, wine, jams, jellies, margarine, meat products, cakes, sauces, processed cheese, salads, etc.	[87]
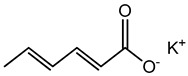 Potassium Sorbate	Contact dermatitis and urticarial, etc.	Karela amla juice, Amla-Aloe vera juice with wheatgrass, milk products, etc.	[87]
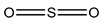 Sulphur dioxide/Sulphites 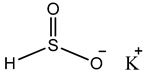 Potassium bisulfate	Allergic reactions (nausea, skin irritation, asthma, eczema, diarrhea)	Fruit juices, cider, wines, meat products, sausages, sweets, jams, jellies, glucose syrups, dry biscuits, dry mix of rasogollas, refined sugar, misri, jaggery, glucose syrup, potatoes, beverages, raisins, pickles, chutneys, etc.	[87]
Nitrites & Nitrates (Sodium & Potassium)	Genotoxicity, carcinogenic (stomach and pancreatic cancer)	Meat products, hot dog, smoked fish, and many others	[87,110]
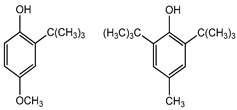 BHA BHT	Endocrine disruptors, neurological problems, metabolic dysfunction, behavioral issues, and may cause cancer	Hot dogs, meat, potato chips, chewing gum, vegetable oils, bread, breakfast cereals, and many others	[111]
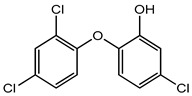 Triclosan	Antibiotic resistance in bacteria, endocrine disruptor, skin sensitization, neuro-toxicity, reproductive and developmental toxicity, genotoxicity, carcinogenicity, phototoxicity, etc.	Active food packaging	[49,111,112]
Nisin	Nausea, pruritus, skin rash and vomiting, etc.	Prepacked coconut water, canned rasgulla, baking mixes containing egg, etc.	[113]
Dehyderoacetic acid (DHA)	Allergic contact dermatitis	Cheese, meat products, squash, etc.	[114]
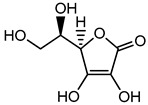 Erythorbic acid	Hypersensitivity, nausea, vomiting, headache, diarrhea, heartburn, and cramps	Meat, frozen fruits and vegetables, beverages such as soft drinks, fruit juice, grape wine, beer, etc.	[115]
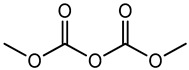 Dimethyl dicarbonate (DMDC)	Carcinogenic	Non-alcoholic beverages such as sports drinks, energy drinks and non-carbonated drinks such as ice tea, etc.	[116,117,118]

**Table 4:** List of existing pharmaceutical preservatives and their problems.

Preservative & Its Structure	Category	Toxic Effect	Pharmaceuticals	References
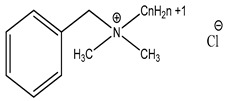 Benzalkonium chloride solution, I.P (BAK) Ocular Preservative	Quaternary ammonium compound	Ocular toxicity, cytotoxicity, redness, dry eye syndrome, nasal mucosal damage, conjunctiva, post-operative inflammation, bacterial resistance, impairs tear film, genotoxicity, trabeculectomy failure, leaching of the eye lens, cataract, etc.	Eye/ear drops, nasal drops, multisurface disinfectants, Dettol, Savlon, Lysol, etc.	[68,122,123,124,125,126]
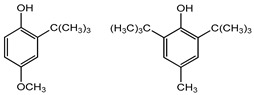 BHA BHT	Phenolic compounds	Gastric irritant, endocrine disruptors, and may cause cancer	Gels, creams, liquid and gelatin capsules	[127,128,129]
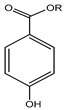 R = an alkyl group Methyl paraben, ethyl paraben, propyl paraben, isobutyl paraben, butyl paraben	Paraben	Sensitization, contact dermatitis, estrogenic effect interferes with male reproductive functions, breast cancer, contact eczema, development of malignant melanoma, etc.	Local anesthetic (bupivacaine hydrochloride injection I.P.), injections (Afkim 500, gentamicin, amikacin sulphate, dexamethasone sodium phosphate injection I.P.), shaving cream, etc.	[130,131]
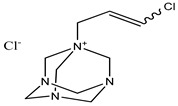 Quaternium-15	Formaldehyde releaser	Sensitization, allergic contact dermatitis	Eye drops, contact solutions	[131,132]
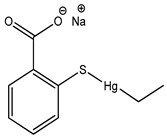 Thiomersal	Mercurial	Genotoxicity, apoptosis, cytotoxicity, neurotoxicity, neurodevelopmental and CVS disorders	Vaccines (inactivated influenza vaccine, meningococcal polysaccharide vaccine, tetanus toxoid, DTP), contact lens solutions, disinfectant (merthiolate),	[65,68,133]
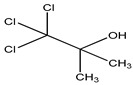 Chlorobutanol	Alcohol	Not used in aerosol dispensers, cytotoxicity, retinal toxic irritation	ophthalmic ointment, ear drops, eye and nasal drops, injections, sedatives, etc.	[65,134]
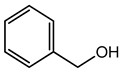 Benzyl Alcohol	Alcohol	Contact dermatitis, mucous membrane irritation, CNS depression, lethargy, metabolic acidosis, toxicity of fatal development, and respiratory abnormalities, etc.	Injectable drugs (anti-inflammatory, neuroleptics), cream, and lotion, etc.	[135,136,137,138]
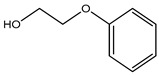 Phenoxyethanol	Glycol ether	Eye and skin irritation, allergic reactions such as contact urticaria and contact dermatitis, etc., are rare.	DTwP vaccines, DTaP vaccine	[139,140,141]
Stabilized Oxychloro complex (second generation ocular preservatives)	Oxidative complexes (soft preservatives)	Cytotoxicity	Eye drops	[68,125]
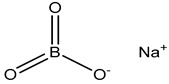 Sodium perborate (GenAqua)				
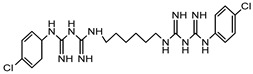 Chlorhexidine	Amidines or biguanides	Ototoxic, nausea, stomach irritation, respiratory distress syndrome	Eye drops, antiseptic mouthwashes	[68,142]
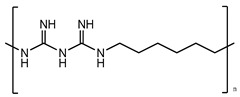 Polyhexamethylene biguanide (PHMB)	Polyhexanide	Carcinogenic, contact allergy, conjunctival staining, corneal staining, pain after instillation, conjunctival hyperemia	Eye drops, contact lens solutions	[143]
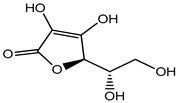 Ascorbic acid	Organic acid	Nausea, vomiting, headache, heartburn, and cramps	Eye drops	[80,81,82]
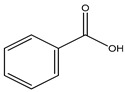 Benzoic acid	Acids and their salts	Carcinogenic, irritation, metabolic acidosis, asthma, convulsions, etc.	Ointments, liquid preparations	[83,84,85]
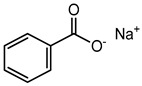 Sodium benzoate	Acids and their salts	Allergy, asthma, skin rashes, hyperactivity, liver cirrhosis, Parkinson’s disease, etc.	Oral suspension, mouthwash, syrup, janam ghunti, etc.	[83,84,85,87]
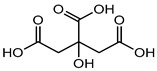 Citric acid	Acids and their salts	Numbness, rapid weight gain, cramps, mood changes, severe stomach pain, diarrhea and convulsions, etc.	Powder and vitamins, etc.	[86]
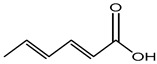 Sorbic acid	Acids and their salts	Contact dermatitis, and urticaria	Emulsions	[87]

**Table 5:** List of some phenolic acids and their reported biological activities.

Natural Sources	Phenolic Content with Antioxidant/Antimicrobial Activity	References
*Allium cepa*	Protocatechuic acid	[161]
*Ananas comosus*	Coumaric acid and ferulic acid	[162]
Berries	Hydroxycinnamic and hydroxybenzoic acids	[163,164,165]
*Coffea arabica*	Hydroxycinammic acids	[164]
*Daucus carota*	*p*-Coumaric, chlorogenic, and caffeic acids	[166,167]
*Emblica officinalis*	1,8-Cineole	[168,169]
*Fagopyrum esculentum*	Rutin, quercetin, and catechins	[170,171,172]
*Glycine max*	Protocatechuic acid, *p*-hydroxybenzoic acids, chlorogenic acid, etc.	[173]
*Malus domestica*	Coumaric acid, catechin, epicatechin, chlorogenic acid, procyanidin and gallic acid, etc.	[174]
*Mangifera indica*	Myricetin, chlorogenic acid, kaempferol, gallic acid, sinapic acid, and ferulic acid	[175]
Mushroom species	*p*-Hydroxybenzoic, protocatechuic, gallic, vanillic, coumaric, syringic, gentisic, ferulic, cinnamic, caffeic acids	[176]
*Origanum vulgare*	Rosmarinic acid	[177]
*Oryza sativa*	Protocatechuic acid	[178]
*Psidium guajava*	Chlorogenic Acid, gallic acid, Kaempferol, sinapic acid, and myricetin	[175]
*Punica granatum*	Quercetin, kaempferol, luteolin, and Myricetin, etc.	[179]
*Salvia rosmarinus*	Rosmarinic acid, carnosol, and carnosic acid	[177]
*Syzygium cumini*	Quercetin	[180,181,182]
*Thymus vulgaris*	Caffeic and rosmarinic acids	[183]
*Triticum aestivum*	Phytic acid, ferulic acid, sinapic, syringic, vanillic and *p*-coumaric acids	[184,185]
*Vitis vinifera*	Malvidin, cyanidin, delphinidin, peonidin and petunidin, epigallocatechin etc.	[186,187]

**Table 6:** List of approved/permitted preservatives with a code assigned by the International Numbering System (INS) and E numbers (EU).

INS Number	E Number	Preservative
200	E200	Sorbic acid
201		Sodium sorbate
202	E202	Potassium sorbate
203		Calcium sorbate
209		Heptyl para-hydroxybenzoate
210	E210	Benzoic acid
211	E211	Sodium benzoate
212	E212	Potassium benzoate
213	E213	Calcium benzoate
214	E214	Ethyl *p*-hydroxybenzoate
215	E215	Sodium ethyl *p*-hydroxybenzoate
216		Propyl *p*-hydroxybenzoate
217		Sodium propyl *p*-hydroxybenzoate
218	E218	Methyl *p*-hydroxybenzoate
219	E219	Sodium methyl *p*-hydroxybenzoate
220	E220	Sulphur dioxide
221	E221	Sodium sulphite
222	E222	Sodium hydrogen sulphite
223	E223	Sodium metabisulphite
224	E224	Potassium metabisulphite
225		Potassium sulphite
226	E226	Calcium sulphite
227	E227	Calcium hydrogen sulphite
228	E228	Potassium hydrogen sulphite
230		Diphenyl
231		Ortho-phenylphenol
232		Sodium ortho-phenylphenol
233		Thiabendazole
234	E234	Nisin
235	E235	Natamycin (Pimaricin)
236		Formic acid
237		Sodium formate
238		Calcium formate
	E239	Hexamethylene tetramine
240		Formaldehyde
241		Gum guaiacum
	E242	Dimethyl dicarbonate
	E243	Ethyl lauryl arginate
	E249	Potassium nitrite
	E250	Sodium nitrite
	E251	Sodium nitrate
	E252	Potassium nitrate
260		Acetic acid, glacial
261		Potassium acetates
262		Sodium acetates
263		Calcium acetates
264		Ammonium acetate
265		Dehydroacetic acid (DHA)
266		Sodium dehydroacetate
270		Lactic acid
280	E280	Propionic acid
281	E281	Sodium propionate
282	E282	Calcium propionate
283	E283	Potassium propionate
	E284	Boric acid
	E285	Sodium tetraborate; borax
290		Carbon dioxide
	E1105	Lysozyme
344		Lecithin citrate
384		Isopropyl citrates
386		EDTA

**Table 7:** Amendments adopted by FSSAI gazette as per 7th Amendment Regulations, 2020.

Food Additive	INS No.	Old Maximum Limits	Recommended Maximum Limits	Inclusion/ Omission
Pimaricin (natamycin)	235			Omitted
Sorbates	200	2000 mg/kg	1000 mg/kg	
EDTA	386	100 mg/kg	50 mg/kg	
Potassium iodate				Omitted
Potassium bromated				Omitted

**Table 8:** New recommended maximum limits for some food additives.

Food Additive	Old Maximum Limits	Recommended Maximum Limits	Inclusion/ Omission
Calcium sorbate			Omitted
Climbazole	0.5%	0.2%	
Hydroxyethoxyphenyl butanone (HEPB)		0.7% *w*/*v*	New entry

**Table 9:** Nanoparticles for food preservation.

Nanomaterial/ Nanoparticles	Definition	Matrix	Applications/Activity	Reference
Ag	Particles of matter fluctuating between 1 and 100 nm in diameter display distinctive properties	Poultry meat, fruits, and vegetables such as apples, grapes, tomatoes, kiwi, asparagus, etc.	1. Antimicrobial agents in food packaging. 2. Preservation of storage containers and refrigerators. 3. As a health supplement. 4. Active food packaging.	[230,236,237,238,239,240]
ZnO		Orange juice, apple juice, peaches, mango, tomato, poultry meat, etc.	1. Antimicrobial agents, as a nutritional additive in food packaging. 2. Increase the shelf life. 3. Enhancing the quality of cucumber, increasing zinc, iron, and carotene content.	[230,241,242,243,244,245,246,247,248,249]
TiO_2_		Strawberry, soft cheese, Chinese jujube, etc.	Food additives for storage containers and food packaging.	[230,237,240,243,245,246]
Silver oxide		Apple slices	Retard microbial spoilage	[230,240,243,247,248]
Nanocomposites	Multiphase materials consist of two or more components in which one of the components has nanoscale dimensions to obtain the best properties of each component	Beans, pear, mushrooms, carrot, cheese, tomatoes, fresh fruits and vegetables, etc.	1. Polylactic acid (PLA)/nano clay/nanocellulose hybris nanocomposite offer a reduction in oxygen transmission rate and water vapor transmission rate. 2. Biodegradable nanocomposite films (dye/clay/PLA) applied in food packaging offers excellent barrier properties.	[249,250,251,252]
Nanoclay (natural nanolayer structures)	Nanoparticles of layered mineral silicates (phyllosilicates) of nanoscale dimensions	Cheese, processed meat, fruit juices, milk products, etc.	1. Intercalation of drugs (such as sildenafil, aripiprazole) in montmorillonite (MMT) used to improve organoleptic properties. 2. Sepiolite clay efficiently applied in active food packaging, enhances antimicrobial and antioxidant properties when incorporated with essential clove oil. 3. By loading Vitamin A in sepiolite (SPT), its oxidative degradation can be prevented. 4. Cefazolin loaded in chitosan/PVA/SPT hybrid hydrogel films showed a wider zone of inhibition against Bacillus cereus bacterium. 5. Drug–clay complex increases the dissolution as well as drug release rate. For e.g., Vitamin B_1_ loaded montmorillonite (MMT).	[252,253,254,255,256,257,258,259]
Nanoemulsions	Nanosized colloidal systems designed to improve drug delivery systems	Encapsulation of bioactive components such as resveratrol, probiotics, nutraceutical, PUFAs, flavored nanoemulsions with improved curcumin digestibility.	1. Encapsulation of lipophilic components such as vitamins. 2. Beeswax–starch emulsion, o/w applied as an edible coating in food preservation. 3. Nanoemulsion powder with turmeric extract, o/w enhances the shelf life of fortified milk for 3 weeks. 4. Sapindus extract and basil oil, o/w with antimicrobial activity against food pathogens. 5. Kemira nanogel enhances skin smoothness. 6. Cumin seed oil, corn oil, whey protein with antifungal activity in food preservation.	[260,261,262,263,264]
DNA biochips, electronic tongue (Nano sensors)	Sensitive device that operates at the nanoscale level to detect and transmit chemical, biological, and physical information to the macroscopic level.	Wine characterization, fruits, meat products	1. To detect pathogens and toxins in foodstuffs. 2. To monitor the freshness of foodstuffs. 3. Incorporated into packaging materials for monitoring product spoilage.	[265,266,267]
Nano edible coating and films	A thin layer of edible materials is applied on the surface of an edible product to preserve it from the external environment.	Bread, frozen food, pizza, cakes, meat products, ice creams, fruits (apple, grapes, papaya, mangoes), potato, tomato, broccoli, etc.	1. To regulate humidity, oxidation, and gaseous exchange to preserve the foodstuffs against invading microbes. 2. As packaging materials. 3. Pathogen inhibitor. 4. Food preservation.	[236,240,268,269,270]

**Table 10:** Multifunctional ingredients with antimicrobial properties.

S.No.	Multifunctional Ingredients	Examples
1	Surfactants	Anionic (Stearic acid) Cationic (cetyl pyridinium chloride) Non-ionic (propylene and ethylene oxide)
2	Fatty acids and esters	Caprylic acid, capric acid, heptanoic acid, etc.
3	Biomimetic phospholipids	Lecithin
4	Antioxidants as preservatives	BHA, BHT, propyl gallate etc.
5	Aroma chemicals as preservatives	Spices and essential oils (oil of rose, lemon, clove, etc.)
6	Chelating agents as preservatives	EDTA, citric acid, phytic acid, etc.
7	Fragrance Ingredients	Benzyl acetate, phenethyl alcohol, linalool

**Table 11:** Legislation on cosmetic products in India, USA, and Europe.

Contents	India	USA	Europe
Governing authority	Central Drugs Standard Control Organization (CDSCO)	Food Drug Administration (FDA)	European Union Regulatory Authority (EUMA)
Rules and regulations	Drugs and Cosmetics Act, 1940, and Rules 1945 Bureau of Indian Standards (BIS)	Food, Drug & Cosmetic Act (FD&C Act)	Council Directive 76/768/EEC, 1976 Revised Regulation EC 1223/2009
Purpose	BIS regulates the standards for cosmetics products described under Schedule “S” of the Drugs and Cosmetics Rules 1945	Enforcement of laws governing the marketing of cosmetics prohibits the marketing of adulterated or misbranded cosmetics in the market	Provides comprehensive categorization of cosmetic products on the basis of function, zone of application, and product constitution
Cosmetics	Any article envisaged to be poured, rubbed, sprinkled, or sprayed on, or introduced into, or otherwise applied to the human body or any part thereof for cleansing, alluring, beautifying, or changing the appearance, and entails any article intended for use as a component of a cosmetic.	Any article envisaged to be poured, rubbed, sprinkled, or sprayed on, or introduced into, or otherwise applied to the human body or any part thereof for cleansing, alluring, beautifying, or changing the appearance, and entails any article intended for use as a component of any such articles; except that such term shall not include soap.	Any substance or preparation intended to be placed in contact with the various external parts of the human body or with the teeth and the mucous membranes of the oral cavity with a view exclusively or mainly to cleaning them, perfuming them, changing their appearance and/or correcting body odors and/or protecting them or keeping them in good condition.
Registration	Required	Through Voluntary Cosmetic Registration Program (VCRP) (Not mandatory)	For market authorization, an applicant must provide a degree of information in the application form.
Pre-market approval	Required under state government licensing	Not required	Not required
GMP compliance	Yes	Yes Not mandatory	Yes ISO 22716 is the reference guide to GMP implementation and assessment
Level of microbial contamination		Follows the guidelines of the Personal Care Products Council (PCPC), formerly the Cosmetic, Toiletry and Fragrance Association (CTFA)	Scientific Committee on Consumer Safety (SCCS) Guideline “SCCS Notes of Guidance for the Testing of Cosmetic Ingredients and their Safety Evaluation, 9th revision”
Post-marketing survey and Reporting system	No specific guidelines	Follows Voluntary Cosmetic Regulation Program (VCRP) reporting system	No specific guidelines
References	[298,299,300]	[24,294,295,296,298,301,302,303]	[24,222,298,299,304,305]

**Table 12:** Legislation on food products in India, USA, and Europe.

Contents	India	USA	Europe
Governing authority	FSSAI	Food Drug Administration (FDA)	European Union Regulatory Authority (EUMA)
Rules and regulations	Food Safety and Standards Act (FSSA), 2006 Bureau of Indian Standards (BIS)	Food, Drug & Cosmetic Act (FD&C Act)	Directive 95/2/EC
Purpose	For laying down standards for food and to regulate their manufacture, storage, distribution, sale, and import, to ensure availability of safe and wholesome food for human consumption and advancement in international trade	FDA’s Centre for Food Safety & Applied Nutrition (CFSAN) have regulatory control by laying down standards and policies to prevent gross adulteration and contamination for ensuring the safety of food	Establishment and effective functioning of the internal market, health, safety, environmental protection, and consumers protection
Features	BIS deals with the standardization of food products and runs a voluntary certification scheme known as the “ISI” mark for processed foods	Authorized three kinds of food standards—identity, quality, and fill with container	Provides scientific advice and technical support for farming legislation and policies having a direct or indirect impact on food and food safety
Additive/ preservative	As per amendment regulation, any substance that is capable of inhibiting, retarding, or arresting the process of fermentation, acidification or other deterioration of food but does not include: any permitted coloring matter; common salt (sodium chloride); lecithin, sugars or tocopherols; nicotinic acid or its amide		Preservatives are described as food additives that are intentionally added to the foodstuff that become a component of the foodstuff, to prolong its shelf life by preventing microbial contamination
Pre-market approval	Required	Required	Required
Food safety & management systems	Good Manufacturing Practices (GMP) Good Handling Practices (GHP) Hazard Analysis Critical Control Points (HACCP)	Require proof of Safety review GRAS is an American Food and Drug Administration (FDA) assignment that a concoction or substance added to food is viewed as protected by specialists	
References	[195,308,309,310,311]	[312,313]	[197,199,314,315]

**Table 13:** Legislation on pharmaceutical products in India, USA, and Europe.

Contents	India	USA	Europe
Governing authority	Central Drug Standard Control Organization (CDSCO) headed by Drugs Controller General of India (DCGI), Ministry of Health and Family Welfare, Government of India	Food Drug Administration (FDA)	European Union Regulatory Authority (EUMEA)
Rules and regulations	Drugs and Cosmetics Act, 1940 and Rules 1945—to regulate import, manufacture, licensing, testing, distribution, and sale of the drug in India. The Pharmacy Act, 1948—to regulate the pharmacy profession in India Drugs and Magic Remedies (Objectionable Advertisement) Act,1954	Food, Drug and Cosmetic Act (FD&C Act)	Directive 2001/82/EC (Veterinary medicinal products) Directive 2001/83/EC (medicinal products for human use)
Purpose	To regulate import, manufacture, distribution, sale, clinical trials, market authorization, and post-market surveillance of drugs in India	To regulate import, manufacture, distribution, and sale of drugs	Provides a legal framework for authorization, manufacture, and distribution of a drug in the EU
Drug	Chemical substance which alters the whole-body function and has the potential for misuse	Defines drugs as “articles intended for use in the diagnosis, cure, mitigation, treatment, or prevention of disease and articles (other than food) intended to affect the structure or any function of the body of man or other animals”	Pharmaceutical preparations are defined as medicinal products consisting of active substances that may contain excipients, formulated into a dosage form suitable for the intended use
Additive/ Preservative	Any chemical or natural substance including fumigants capable of inhibiting or retarding the microbial growth	Any inactive fixings that are purposefully added to the therapeutic and diagnostic items, however it is not anticipated that they would apply remedial impacts at the planned dosage, regardless of the way that they may act to improve item delivery that presents well-being data concerning the correct and now proposed level of introduction, exposure time, or route of administration	Novel additives are described as the substances that are introduced for the first time in a formulation in addition to the active drug component or may be administered through a new route administration New additives are treated as a new entity or drug in the EU
Licensing/ Authorization	Required	Required	Required
Safety & management systems	Schedule M—GMP and requirement specifications of factory premises, plant and equipment for pharmaceutical products. Schedule T—GMP specifications for the manufacture of Ayurveda, Siddha, and Unani medicines. Schedule Y—specifications for clinical trials, import, and manufacture of new drugs	FDA issued guidelines for new additives entitled “Non-Clinical Studies for the Safety Evaluation of Pharmaceutical Excipients” to establish the safety of dosage forms	IPEC Europe issued guidelines for evaluating the safety of new additives entitled “Guideline on additives in the dossier for application for marketing authorization of a medicinal product”. Documentation on every single novel additive is required on the principle of the CPMP Guideline (Chemistry of New Active Substances)
Regulatory aspects	GCP guidelines in accordance with WHO guidelines and ICH requirements for good clinical practices	FDA takes ICH safety testing direction papers for leading safety tests	European Medicines Agency (EMA) has established that pharmaceutical companies must follow GMP procedures to ensure the quality standards of medicinal products
References	[317,318]	[319,320]	[321,322,323]

## Data Availability

Not applicable.
